# Fluoride release and recharge from bioactive resins *in vitro*

**DOI:** 10.4317/jced.63076

**Published:** 2025-10-01

**Authors:** Antônio Igor Figueira da Silva, Lukas Herycles do Nascimento Santos, Marcus Vinícius Oliveira, Lyzia Vitoria Mendes Rezende, Glauber Campos Vale

**Affiliations:** 1 Department of Restorative Dentistry, Federal University of Piaui, Teresina, Piauí, Brazil

## Abstract

**Background:**

Bioactive resins can release fluoride ions, but their potential for fluoride recharge is unknown. This study aimed to evaluate the fluoride (F) release and recharge capacity of bioactive resins using an *in vitro* pH cycling model.

**Material and Methods:**

Six specimens were prepared for each group: two bioactive resins (Beautifil Flow Plus and Biocoat), a conventional resin (Opallis Flow), and a resin-modified glass ionomer cement (Ionoseal). For pH cycling, each specimen was immersed in an acidic solution (Sprite, pH 3.6) for 6 hours at 37 °C, rinsed with distilled water, and then stored in artificial saliva (pH 7.0) for 18 hours. This cycle was repeated for three consecutive days. Afterward, the samples were brushed with fluoride toothpaste and subjected to a second pH cycling phase. For F determination, 1 mL of TISAB was added to each solution, and fluoride levels were measured using an ion-selective electrode. Data were tested for normality using the Shapiro–Wilk test and analyzed by one-way ANOVA followed by Tukey’s post hoc test.

**Results:**

In the initial fluoride release phase, Beautifil resin demonstrated similar performance to Ionoseal and exhibited a significantly higher fluoride release compared to the other materials (*p* < 0.05). However, during the recharge phase, Ionoseal demonstrated superior fluoride release compared to all other materials (*p* < 0.05).

**Conclusions:**

Beautifil resin and Ionoseal exhibited the highest fluoride release in both phases of pH cycling when compared to the other materials. Nevertheless, Ionoseal outperformed the others in fluoride recharge following treatment with fluoridated dentifrice.

** Key words:**Fluorides, Bioactive Resin, Dental Materials.

## Introduction

Fluoride exerts its protective effect through its physicochemical ability to inhibit demineralization and promote remineralization, leading to the precipitation of fluorapatite on tooth surfaces. Therefore, the use of fluoride, when present in its free and soluble form in an aqueous medium, is an effective measure for controlling dental caries [1-15].

In addition to various delivery methods, several restorative dental materials can serve as supplementary sources of fluoride release [5,15]. The availability of active fluoride ions depends on factors such as the composition of the material’s matrix, its bonding mechanism, fluoride content, and environmental conditions [16-19]. Fluoride at the tooth–restoration interface helps prevent caries progression and contributes to the longevity of restorations [4,5].

Materials capable of facilitating the incorporation of minerals like fluoride into the dental substrate are classified as bioactive [18]. Among them, glass ionomer cement (GIC) is considered the most effective bioactive material due to its high potential to promote remineralization of dental tissues. However, its mechanical and aesthetic limitations are often highlighted in comparison to composite resins [7,12].

In response, composite resins have evolved to include therapeutic functions in addition to their inherent physical and aesthetic qualities. Bioactive composite resins, introduced over 15 years ago, incorporate pre-reacted glass ionomer particles into a resin matrix [10,15]. This composition allows the release of fluoride ions and supports the remineralization of dental structures adjacent to the restoration, reducing the risk of secondary caries [7].

From this perspective, it is known that these materials can release fluoride ions, raising interest in their potential for fluoride recharge, given that cariostatic fluoride concentrations may decrease over time [8]. Fluoride recharge can be achieved through external sources such as fluoridated toothpaste or professional fluoride treatments, aiming to sustain optimal ion availability [14]. However, scientific evidence supporting the clinical effectiveness of this strategy remains limited.

## Material and Methods

- Specimen Preparation

A comparative laboratory study was conducted using two bioactive resins: Beautifil Flow Plus (SHOFU Inc., Kyoto, Japan) and Biocoat Bioactive (Premier), alongside a non-bioactive composite resin (Opallis Flow, FGM) and a resin-modified glass ionomer cement (Ionoseal, Voco). For each material group, six specimens were prepared in accordance with NBR ISO 4287 (International Organization for Standardization). The dimensions of each specimen were standardized at 4 mm in length, 4 mm in width, and 2 mm in thickness. The materials were prepared and handled in a controlled laboratory environment at a temperature of 26 ± 1 °C. Each restorative material was inserted into a silicone mold in a single increment using a spatula and light-cured for 40 seconds. Excess material was removed using tweezers, and each specimen was numbered.

- pH Cycling and Fluoride Dentifrice Treatment

Each specimen was individually placed in a culture plate containing 1 mL of an acidic solution (Sprite®, pH 3.6) and incubated at 37°C for 6 hours. After this period, the acidic solution was replaced, and the specimens were rinsed with distilled water and immersed in 1 mL of artificial saliva at 37°C for 18 hours. This demineralization–remineralization cycle was repeated for three consecutive days. Following the initial pH cycling, the specimens were treated with a slurry (1:3 weight/volume) of a conventional fluoride toothpaste containing 1450 ppm F for 5 minutes, rinsed with distilled water, and then subjected to a second round of pH cycling following the same protocol.

- Fluoride Release Measurement

To quantify fluoride release, 0.1 mL of TISAB III (Total Ionic Strength Adjustment Buffer) was added to each well containing the acidic or saliva solution to ensure complete fluoride ion dissociation. The resulting solutions were analyzed using a fluoride ion-selective electrode (Orion 9606 – Orion Research Inc., USA) connected to a potentiometer. Measurements were performed under continuous agitation using a magnetic stirrer. Prior to analysis, a calibration curve was established using standard solutions ranging from 0.025 to 4.0 ppm fluoride.

- Statistical Analysis

Data normality was confirmed by the Shapiro–Wilk test. A two-way ANOVA was conducted to evaluate the effects of material type and treatment condition (before and after fluoride exposure) on fluoride release. Tukey’s post hoc test was used for multiple comparisons among groups. Statistical analyses were performed using GraphPad Prism (version 10.0 for Windows), with the significance level set at 5% (*p* < 0.05).

## Results

 Table 1 presents the fluoride release and recharge values for the tested materials across the evaluation periods. Beautifil resin and Ionoseal demonstrated the highest levels of fluoride release (*p* < 0.05) compared to the other materials, with no statistically significant difference between them (*p* > 0.05). Regarding fluoride recharge, Ionoseal exhibited the greatest release following exposure to the fluoridated dentifrice, followed by Beautifil. For most materials, no significant differences in fluoride release were observed across the different time points. An exception was Beautifil resin, which showed significantly higher fluoride release and recharge on the first day compared to the subsequent days (*p* < 0.05). Both Biocoat and Opallis resins demonstrated similar and comparatively lower fluoride release levels, despite Biocoat being classified as a bioactive material.

**Figure 1:**
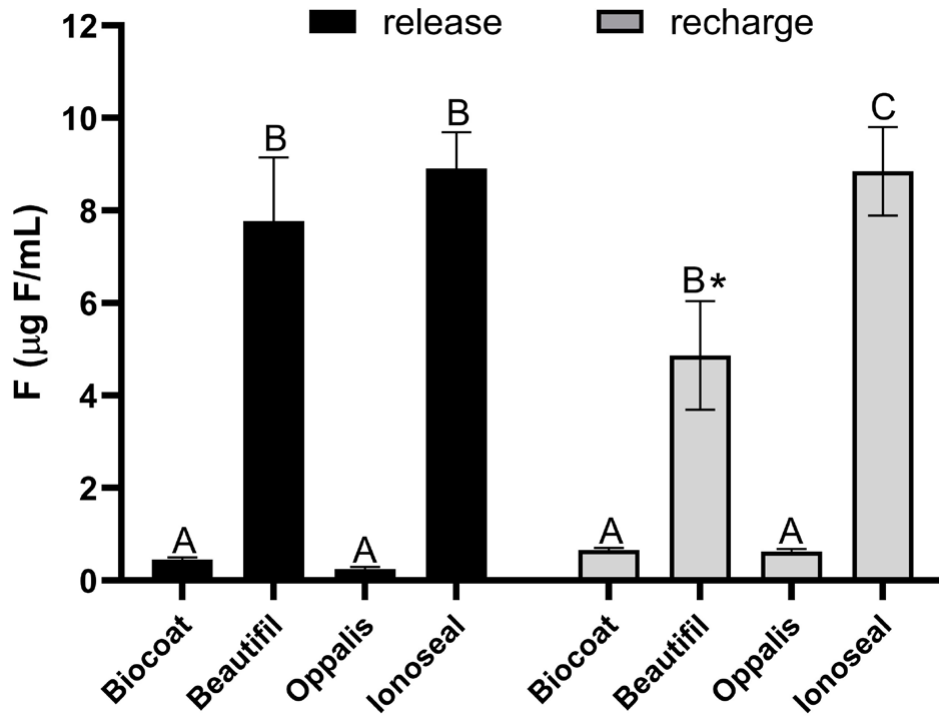
Cumulative fluoride release and recharge after pH cycling according to the resins studied (n=6). Different letters indicate a statistically significant difference between the resins (*p* < 0.05); the asterisk indicates a statistically significant difference in resin between release and recharge (*p* < 0.05). The vertical bars indicate the standard deviation.

Figure 1 illustrates the cumulative fluoride release and recharge after pH cycling. Following the initial pH cycling, Beautifil and Ionoseal released significantly more fluoride than Biocoat and Opallis (*p* < 0.05), with no significant difference observed between the latter two. After fluoride dentifrice treatment and a second round of pH cycling, Ionoseal again showed the highest fluoride release (*p* < 0.05), followed by Beautifil. Biocoat and Opallis continued to show no significant differences between them and maintained the lowest fluoride release and recharge levels throughout the study. Notably, Beautifil resin exhibited a decrease in fluoride release following the recharge phase compared to its initial release (*p* < 0.05).

## Discussion

Fluoride-releasing dental materials have proven effective in promoting the remineralization of tooth structures adjacent to restorations [7]. Conventional glass ionomer cements (GICs) are recognized for their superior bioactivity compared to composite resins, primarily due to their higher fluoride release and intrinsic acid–base setting reaction [3,9]. However, in the present study, Beautifil resin exhibited fluoride release levels comparable to those of Ionoseal (resin-modified GIC) during the initial phase, prior to exposure to fluoridated dentifrice.

Bioactive resins are designed to combine esthetic, mechanical, and biological properties, particularly under acidic conditions [2,20], making them suiTable for patients with high caries risk [9,15]. Their cariostatic potential is closely linked to both the amount and duration of fluoride release [13]. In this study, Beautifil resin showed a significantly higher fluoride release on the first day, underscoring a pronounced initial effect. This early fluoride burst may reduce bacterial viability, inhibit cariogenic activity, and promote remineralization [3].

The initial “burst effect” of fluoride release within the first 24 hours is a well-documented feature of glass ionomers [2], and in this study, a similar pattern was observed for both Ionoseal and Beautifil resin. In contrast, Biocoat, despite being marketed as a bioactive resin, showed fluoride release and recharge levels similar to those of the conventional resin Opallis. This outcome may be attributed to differences in resin matrix composition and fluoride-binding mechanisms [11].

All tested materials were capable of recharging fluoride following treatment with fluoridated dentifrice, although to varying extents. As expected, and supported by the literature [3,9,13], Ionoseal demonstrated the highest fluoride recharge capacity. This performance is likely due to its porosity and permeability, which promote greater fluoride diffusion and interaction with glass particles [9]. It is important to note that fluoride re-release after recharge is generally lower than the initial release [6], as also observed with Beautifil resin, which exhibited reduced fluoride output following the recharge phase. This pattern is consistent with previous findings on fluoride-releasing composites [6].

Despite the similar initial fluoride release between Beautifil and Ionoseal, the latter showed superior recharge performance after exposure to a conventional dentifrice containing 1450 ppm F. This finding contrasts with studies in which Beautifil exhibited recharge capabilities comparable to nanoglass ionomers when subjected to higher fluoride concentrations (5000 ppm F) [9]. Therefore, the fluoride concentration in the recharging medium plays a critical role in determining re-release potential [3].

Fluoride uptake and subsequent release are also influenced by the material’s permeability; more impermeable materials tend to adsorb ions only superficially. This may explain the slight increase in fluoride release observed for Beautifil on the first day after recharge, likely due to the leaching of surface-adsorbed ions and the presence of unbound fluoride within the resin matrix [19].

Despite efforts to simulate the dynamic pH conditions of the oral environment, the limitations of this *in vitro* study must be acknowledged, as the results may not fully reflect clinical performance. Therefore, *in vivo* studies are essential to validate these findings. Nonetheless, the present results provide valuable insights that may assist clinicians in selecting restorative materials based on the specific preventive needs of individual patients.

## Conclusions

Beautifil resin exhibited fluoride release levels comparable to those of the resin-modified glass ionomer cement (Ionoseal) during the initial phase. However, following fluoride recharge, its fluoride release declined relative to the initial output. Biocoat resin, despite being marketed as a bioactive material, did not demonstrate effective fluoride release or recharge capacity and performed similarly to the conventional composite resin Opallis.

## Figures and Tables

**Table 1:** Mean (SD) of fluoride release (ug F/ml) from the resins studied before (release) and after (recharge) treatment with fluoridated toothpaste according to the days (n=6).

Release					Recharge			
Time	Biocoat	Beautifil	Oppalis	Ionoseal	Biocoat	Beautifil	Oppalis	Ionoseal
Day 1	0.12 (0.02) aA	3.61 (1.24) bA	0.05 (0.01) aA	3.28 (1.55) bA	0.22 (0.03) aA	2.36 (0.68) bA*	0.17 (0.02) aA	3.06 (0.39) cA
Day 2	0.17 (0.05) aA	2.17 (1.14) bB	0.08 (0.02) aA	3.27 (0.74) bA	0.24 (0.04) aA	1.22 (0.33) bB*	0.23 (0.02) aA	2.96 (0.17) cA
Day 3	0.16 (0.02) aA	2.00 (1.24) bB	0.13 (0.02) aA	2.35 (0.29) bA	0.20 (0.01) aA	1.30 (0.28) bB	0.23 (0.05) aB	2.84 (0.61) cA

## Data Availability

The datasets used and/or analyzed during the current study are available from the corresponding author.
